# Harnessing Hemp (*Cannabis sativa* L.) Seed Cake Proteins: From Concentrate Production to Enhanced Choux Pastry Quality

**DOI:** 10.3390/foods14040567

**Published:** 2025-02-08

**Authors:** Tatiana Capcanari, Eugenia Covaliov, Cătălina Negoița

**Affiliations:** Department of Food and Nutrition, Technical University of Moldova, 9/9 Studentilor St., MD-2045 Chisinau, Moldova; eugenia.covaliov@toap.utm.md (E.C.); catalina.cerchez@toap.utm.md (C.N.)

**Keywords:** oil seed cake, hemp, protein concentrate, amino acid, sustainability, choux pastry

## Abstract

This study explores the production and valorization of hemp seed cake protein concentrate (HPC) as a functional ingredient to enhance the nutritional quality and sensory attributes of choux pastry products, specifically éclairs. By integrating varied concentrations of HPC (0%, 1%, 5%, 10%, 15%, and 20%) into traditional formulations, the physicochemical properties, proximate composition, amino acid profile, and sensory characteristics of the resulting pastries were assessed. Sensory attributes were assessed using the check-all-that-apply (CATA) method, where a trained panel selected applicable descriptors from a predefined list. Results indicated that the incorporation of HPC significantly increased protein content from 8.23% in the control sample (HPC0%) to 11.32% in the HPC20% formulation and improved moisture retention, leading to greater exterior and interior éclairs volume, increasing from 42.15 cm^3^ to 51.5 cm^3^ and from 18.34 cm^3^ to 38.47 cm^3^, respectively. Furthermore, sensory evaluation revealed pronounced differences in attributes such as flavor, appearance, and mouthfeel, with optimal sensory profiles noted at 10% HPC inclusion. The amino acid analysis demonstrated a balanced composition, particularly of essential amino acids, emphasizing HPC’s potential as a valuable protein source, with significant contributions from leucine (8.17 g/100 g protein), isoleucine (5.56 g/100 g protein), and phenylalanine (6.31 g/100 g protein), as well as notable levels of immunoactive amino acids such as arginine (10.92 g/100 g protein) and glutamic acid (20.16 g/100 g protein). These findings highlight the significant nutritional benefits of HPC enrichment, supporting the development of healthier bakery products and contributing to sustainable food practices within the industry.

## 1. Introduction

Nowadays, one of the major contributors to the world agricultural sector, the oilseed industry, supplies a large amount of important oils and byproducts that are basic fundamental components in our daily life [1,2]. This industry is also one of those generating enormous quantities of wastes, mainly seed cakes [3,4,5,6], that in many cases remain underutilized despite their nutritional potential. However, these byproducts can be effectively valorized in the circular economy, contributing to resource efficiency and sustainability by decreasing the overall carbon footprint [7,8,9]. This contributes not only to environmental stewardship but also supports the development of novel food applications responding to the growing consumer demand for sustainability in food production [10,11].

The problem of increasing global protein deficiency has come to the fore as a major concern in modern nutrition [12,13]. Protein is an indispensable macromolecule in various biological functions [14,15,16]. While its value can be acknowledged, access to high-quality protein is inhomogeneously spread across people due to socio-economic status and lack of awareness about nutritional requirements [17]. Therefore, it is common that qualitative protein deficiency, with its burdensome health consequences, affects vulnerable populations, especially in the low-resource settings [18]. Integrating high-quality protein-rich foods into existing dietary patterns can enhance nutritional outcomes [19]. Research indicates that certain agricultural crops hold potential as valuable sources of essential nutrients, particularly in improving the overall quality of dietary protein [20,21,22,23]. As the demand for sustainable and ethical food choices increases, there is a growing shift from traditional animal-based proteins toward plant-derived alternatives, driven by environmental concerns, health benefits, and dietary preferences [24].

Among plant protein alternatives, hemp seed cake represents a promising candidate for food valorization due to its high protein content, balanced amino acid profile, and favorable digestibility, making it particularly suitable for enhancing the nutritional quality of new food matrices [25,26,27]. Unlike other oilseed cakes, hemp seed cake requires minimal processing and has significant environmental and economic benefits when repurposed into food formulations [28,29]. By utilizing this protein-rich residue, sustainability can be improved while unlocking new functional applications in the food sector [30,31]. As a byproduct of hemp oil extraction, hemp seed cake is also rich in dietary fiber, making it a valuable feedstock for functional food formulations [32,33,34,35]. Although hemp seed cake has been incorporated into bread, pasta, and plant-based beverages [4,22,35,36], its application in laminated and aerated pastry products, such as choux pastry, remains largely unexplored. While previous studies have analyzed the chemical composition of hemp seed proteins [25,26], there are limited data on their physicochemical and sensory effects in complex food matrices. Understanding the functional role of hemp seed cake protein concentrate (HPC) in pastry formulations is essential for optimizing its integration into bakery products without compromising technological and sensory quality. Thus, fortifying choux pastry dough with HPC represents an innovative approach in the food industry, offering the potential to enhance the nutritional profile and functional properties of this classic pastry product. This research aims to explore the potential of hemp seed cake as a source of protein concentrate and its application in improving the nutritional value and quality characteristics of choux pastry. The study specifically assesses the impact of hemp protein concentrate (HPC) on key physicochemical and sensory attributes of the final product using standardized analytical methods.

## 2. Materials and Methods

### 2.1. Materials

The all-purpose wheat flour, type 550, used in the choux pastry formulation was purchased from the local market. The hemp (*Cannabis sativa* L.) seed cake used in this study was obtained from the local enterprise “Mira OSF” SRL. The seeds belonged to the Secuieni Jubileu variety, a monoecious strain developed in Romania, primarily cultivated for high-nutritional-value oil production. This variety is characterized by a 33.8% oil content, early maturation, and a shorter stem height (1.5–2 m), making it well-suited for mechanized harvesting. Our previous studies have revealed the proximate composition of hemp seed cake: moisture content of 8.24 ± 0.11%, ash content of 6.21 ± 0.05% dry matter (d.m.), total protein content of 31.62 ± 0.22% (d.m.), fat content of 8.19 ± 0.13% (d.m.), and total dietary fiber content of 43.76 ± 0.34% (d.m.) [22]. The formulation of choux pastry also incorporated additional raw materials, including milk with 3.5% fat, butter with 82.5% fat, salt, sugar, table eggs, and drinkable water.

### 2.2. Methods

The acidity, moisture, and ash content of all-purpose (premium quality) wheat flour and choux pastry were analyzed following standardized procedures outlined in international AACC methods (02–31.01 for acidity, 44–15.02 for moisture content, and 08–16.01 for ash content) [37], and the results were compared to the limits set by national regulations [38]. The proximate composition of hemp seed protein concentrate was evaluated using standard AOAC methods [39]. In particular, the moisture content was assessed by AOAC 930.15, crude fat by AOAC 2003.05, crude fiber by AOAC 978.10, and ash content by AOAC 942.05. The crude protein content was calculated from the total proteinogenic amino acids. All analyses were performed in triplicate.

#### 2.2.1. Hemp Seed Protein Concentrate Extraction Procedure

Hemp seed cake powder was prepared by grinding the hemp seed cake using a commercial Kenwood grinder (Chef XL titanium, type KVL80, Kenwood Limited, Havant, UK) and then manually sifting the ground material through a sieve with 200 μm openings. Before the extraction, the powdered cake underwent fat extraction using hexane (99.9% pure, Sigma-Aldrich, Steinheim, Germany) as a solvent, in order to extract a large amount of the lipids from the cake. In more detail, the cake was defatted using a 1:4 ratio (cake–hexane) and soaked for 24 h. The solvent was then removed by evaporation to dryness using a vacuum pump. To ensure the complete removal of residual hexane, the dried samples underwent an additional drying step at 50 °C for 24 h in a ventilated oven, followed by a forced volatilization process under controlled conditions. The absence of residual solvent was verified by monitoring weight stability after drying and by performing an olfactory assessment to detect any remaining volatile compounds. These measures ensured the safety of the obtained flour for food applications. The powdered defatted cake was then mixed with a saline solution of 0.1% *w*/*v*. Based on experimental optimization, the optimal cake-to-solution ratio for maximum protein extraction was determined to be 1:10. The mixture was gently stirred in a laboratory shaker (Orbital Shaker, BTLab Systems, St. Louis, MO, USA) for 30 min at room temperature 21 ± 2 °C to extract the soluble proteins.

Following extraction, the pH of the solution was measured and adjusted to a range of 6.5–7.0 using a 1% acetic acid solution as needed. The obtained mixture was filtered through a Whatman No. 1 laboratory filter paper to remove the solid residues. The filtrate collected in a centrifuge tube was centrifuged at 4000 rpm in a Hettich EBA 20S centrifuge (Hettich, Tuttlingen, Germany) for 15 min, at the end of which the proteins had precipitated.

After centrifugation, the supernatant was removed. The precipitate from the bottom of the tube was washed with distilled water to remove any residual salts and impurities. Subsequently, the high purity of the protein concentrate was assured with a second centrifugation step. Then, the washed precipitate was allowed to dry in a SLN75 drying oven set at 50 °C (POL Ekoaparatura, Wodzisław Śląski, Poland), for 24 h, to obtain the dry hemp cake protein concentrate. The final product was then analyzed for its respective chemical composition: total protein content, carbohydrates, total dietary fiber, fat, moisture, and ash content.

#### 2.2.2. Amino Acid Composition

The amino acid composition in the obtained protein concentrate from hemp seed cake was performed according to the method of Dudok et al. (1997) [40] by acid hydrolysis of proteins into constituent amino acids, with HCl (6N) at 105 °C for 24 h. Further, the amino acids were analyzed on the AAA 339 “Mikrotechna” analyzer (Prague, Czech Republic).

#### 2.2.3. Amino Acid Score

The amino acid score (*AAS*) of hemp seed cake protein concentrate was determined according to the Food and Agriculture Organization using the formula [41]:

(1) $$AAS=\frac{Amino\;acid\;in\;test\;protein\;\left(g\right)}{Amino\;acid\;in\;reference\;protein\;\left(g\right)\;}$$

where the “amino acid in test protein” refers to the concentration of each essential amino acid (*EAA*) in the hemp seed cake protein concentrate (HPC), as determined by amino acid analysis, while the “amino acid in reference protein” corresponds to the FAO/WHO-recommended amino acid requirements for human nutrition, specifically using the reference pattern established for adults [42].

#### 2.2.4. Choux Making Procedure

In this study, choux pastry for éclair production was formulated by incorporating varying concentrations of hemp protein concentrate (HPC) in wheat flour. The experimental design included six formulations, where wheat flour was substituted for HPC at concentrations of 0%, 1%, 5%, 10%, 15%, and 20%. The formulation with 0% HPC was designated as the control sample, as outlined in Table 1.

The choux pastry dough was prepared following the classical methodology defined by Ferrandi School of Culinary Arts (2017), as a way of consistency with the traditional techniques [43]. This was made through very cautious procedural steps in the process, including proper mixing of ingredients, proper temperature during cooking, and timing to arrive at the best consistency of dough. The choux dough was carefully pipetted onto baking trays to form éclair shapes (standard length 12 cm).

#### 2.2.5. Check-All-That-Apply (CATA) Sensory Analysis

The check-all-that-apply (CATA) methodology [44] was employed to evaluate the sensory attributes of the éclairs across various dimensions such as appearance, color, aroma, texture (interior and exterior), aftertaste, mouthfeel, and emotions. A group of 19 trained panelists participated in sensory evaluation. Each panelist was provided with a list of attributes categorized under each dimension as indicated in Table 2. The selection of CATA terms was based on a combination of the existing literature and preliminary sensory evaluation. Initially, descriptors commonly used in pastry sensory analysis were reviewed, followed by a preliminary session with the trained panelists to refine the attribute list, ensuring relevance to the specific characteristics of the tested éclairs.

The sensory analysis was conducted within 24 h after the éclairs were baked, ensuring that all samples were evaluated at optimal freshness and under controlled conditions. The éclairs were presented at room temperature (21 ± 2 °C) in a well-lit, ventilated sensory laboratory, with each panelist receiving identical sample portions on neutral-colored plates to minimize bias. During the tasting session, panelists were instructed to taste each sample and then select all attributes that they felt applied to that particular éclair sample for each category. Panelists evaluated each attribute using a binary scoring system, where a value of 1 (true) was assigned if the attribute applied to the product, and 0 (false) if it did not. After the tasting session, attributes that were not frequently identified by the panelists were eliminated from further analysis. Additionally, based on Cochran’s Q test, characteristics that did not statistically differentiate the samples were filtered out, providing a more accurate representation of the sensory profiles and consumer preferences for the various formulations tested.

#### 2.2.6. Baking Loss

To evaluate the baking loss, the weight of each éclair was measured both before baking and after baking. Baking loss expressed the percentage difference that occurred between the initial weight of the dough and the final weight of the baked éclair [45]. This measure was important, as it refers to the loss of water and volatile compounds during baking and can be used as an indication of baking efficiency and uniformity (Equation (2)).

(2) $$BL\%=\frac{m_{dough}-m_{é\mathrm{clair}}}{m_{dough}}\cdot 100\%,$$

where *BL* is the baking loss value, %;

*m_dough_* is the mass of the dough, g;
  *m_éclair_* is the mass of the baked éclair, g.

#### 2.2.7. Éclair Volume

The total volume of the éclairs, encompassing the external dimensions, was determined using the AACC 10-05.01 rapeseed displacement method [46]. Each éclair was submerged in a graduated cylinder containing a known volume of rapeseeds, and the change in volume was recorded to measure the external volume accurately.

For the internal cavity volume, which represents the space available for fillings, a slightly different approach was employed. Each éclair was carefully cut open, and the internal cavity was filled with rapeseeds. The volume of the seeds used to fill the cavity was measured, providing an accurate assessment of the internal volume.

#### 2.2.8. Water Activity

The water activity of the baked products was measured using a Rotronic water activity meter (INSTRUMART, Williston, VT, USA) [47].

#### 2.2.9. Color Parameters

A CIE Lab* colorimeter CR-400 (Konica Minolta, Osaka, Japan) was used to determine the color [48] of both the external crust and interior of the baked éclairs. For the crust, measurements were taken at multiple locations on the surface of each éclair to capture any color variability. For the interior color assessment, the éclairs were carefully sliced, and measurements were taken at different points inside to obtain a detailed color profile. The L* value indicated lightness, whereas higher values denote a lighter color. The a* value represented the red–green spectrum, with positive values indicating a shift towards red, while the b* value corresponded to the blue–yellow spectrum, with positive values indicating a shift towards yellow. In addition to evaluating the individual color parameters, the color difference, denoted as Δ*E*, was calculated to quantify the overall color change in both the external crust and the interior of the baked éclairs (Equation (3)).

(3) $$\Delta E=\sqrt{{\left(L_{sample}-L_{0}\right)}^{2}+{\left(a_{sample}-a_{0}\right)}^{2}+{\left(b_{sample}-b_{0}\right)}^{2}}$$

#### 2.2.10. Statistical Analysis

All experiments were performed in triplicate, and data were reported as the mean ± standard deviation (SD). Significant differences between the samples were analyzed using analysis of variance (ANOVA) at a 5% level of significance. CATA data analysis was performed using multiple methods. Cohran’s Q test was used to test the independence of products and attributes. All analyses were conducted with XLStat software, version 7.5.2 for Excel.

## 3. Results

### 3.1. Characteristics of Wheat Flour and Hemp Seed Cake Protein Concentrate

The physicochemical properties of wheat flour and hemp seed cake protein concentrate play an important role in determining their nutritional values and potential applications in food products [49]. For wheat flour, the quality assessment focused on moisture, acidity, and ash content, with protein, carbohydrates, and fat levels taken from the product label. In contrast, all relevant physicochemical parameters for the hemp seed cake protein concentrate—including moisture, ash, acidity, total protein, carbohydrates, dietary fiber, and fat content—were assessed directly. The values of these quality indicators are presented in Table 3.

The physicochemical analysis of wheat flour reveals values consistent with national regulations [38], predominantly characterized by high starch content and modest protein levels, which limits its nutritional potential due to a lack of complete proteins and essential amino acids. In contrast, the hemp seed cake protein concentrate exhibited a remarkable total protein content of 74.15% d.m., indicating its potential as a superior protein source for fortifying baked products. This is corroborated by various studies [27,50]; for instance, Niccum (2021) reports that hemp protein isolates can exceed 75.8% protein content and significantly enhance the nutritional quality of baked goods [51]. Additionally, given its substantial dietary fiber content (18.47%), the obtained HPC can further enrich the nutritional profile of choux pastry. According to the literature sources, hemp protein demonstrates superior bioavailability, with digestibility rates between 88% and 91% [52], significantly outpacing that of traditional sources like soybean, which shows digestibility rates ranging from 20.58% to 50.21% depending on the processing method [53]. Additionally, Liener (1994) notes that while soybeans possess high nutritional value, they also contain anti-nutritional factors such as trypsin and chymotrypsin inhibitors, which impede protein digestion and overall nutrient absorption in the intestine [54]. This high digestibility and the presence of essential amino acids highlight the potential of fortifying wheat flour with hemp protein concentrate to yield a nutritionally enhanced pastry with an improved amino acid profile and functional properties, making it a viable option for health-conscious consumers. Overall, integrating hemp protein concentrate not only increases the protein content but also enhances the structural properties of choux pastry dough. Proteins contribute to dough extensibility and expansion by forming a network that traps steam during baking, allowing for volume increase. Additionally, HPC provides functional benefits such as improved emulsification and foaming capacities, which further support the stability and texture of the final product [55]. In the same regards, the reduced fat and moisture content in HPC influences key textural parameters. Lower fat levels (0.45% in HPC vs. 1.08% in wheat flour) may alter aeration and tenderness, as fats facilitate dough expansion and softness, while different moisture levels may affect water absorption, modifying dough viscosity and gelatinization. Although HPC has a lower fat and moisture content than wheat flour, no adjustments were made to the formulation’s water or fat content. This approach was chosen to assess the direct impact of HPC incorporation on choux pastry without altering the standard recipe. Any observed changes in texture, volume, or sensory attributes are thus attributable solely to the presence of HPC rather than external formulation modifications.

After obtaining the protein concentrate from hemp seed cake the total amino acid content in 1 g of protein from the concentrate was determined (Table 4). This analysis highlights the contribution of both essential and non-essential amino acids, as well as other functional amino acid categories.

The total amino acid content in the hemp seed cake protein concentrate reveals a well-balanced composition, particularly in terms of essential amino acids. According to Table 4, the concentrate provides significant amounts of leucine (8.17 g/100 g protein), isoleucine (5.56 g/100 g protein), and phenylalanine (6.31 g/100 g protein), which are key components for protein synthesis and muscle maintenance [56]. Leucine, in particular, plays a central role in activating the mammalian target of rapamycin (mTOR) pathway, which is critical for muscle protein anabolism and recovery, making it especially beneficial for athletes and aging populations at risk of sarcopenia [57]. Methionine and cysteine, sulfur-containing amino acids essential for metabolic functions [58], were present at lower concentrations (1.57 g and 0.44 g/100 g protein, respectively), indicating a possible area for further enrichment if targeting sulfur amino acid deficiencies. Methionine is also a precursor for S-adenosylmethionine, which is involved in methylation processes, while cysteine contributes to glutathione synthesis, a key antioxidant that helps protect against oxidative stress and cellular damage [59]. The results obtained results align with the values reported by Malomo & Sunday et al. (2015), who assessed the amino acid content of commercially available hemp protein concentrates [50].

When analyzing the amino acid score, some amino acids in the hemp seed cake protein concentrate, such as threonine (*AAS*: 234.04%) and isoleucine (*AAS*: 185.26%), significantly exceed the recommended levels. However, others, like cysteine (*AAS*: 19.84%) and valine (*AAS*: 70.57%), fall below the standard, suggesting the need for complementary protein sources in food formulations. The hemp seed cake protein concentrate is also rich in immunoactive amino acids, such as arginine (10.92 g/100 g protein), glutamic acid (20.16 g/100 g protein), and glycine (4.81 g/100 g protein), which play important roles in immune function and inflammation modulation [60,61]. More than that, the concentration of glycogenic amino acids, including alanine (5.98 g/100 g protein) and proline (4.29 g/100 g protein), supports energy metabolism, particularly during gluconeogenesis [62,63]. On the other hand, ketogenic amino acids, such as leucine and lysine, contribute to ketone body formation [64], making the concentrate potentially beneficial in low-carbohydrate or ketogenic diets. These findings align with the research by Xu et al. (2013) [65], which suggests that replacing standard dietary proteins with ketogenic amino acids (*KAAs*) could be a novel strategy to combat hepatic steatosis and other metabolic abnormalities. In the same regard, Zhang et al. (2007) [66] demonstrated that increasing dietary leucine intake reduces diet-induced obesity and improves glucose and cholesterol metabolism in mice via multiple mechanisms, highlighting leucine’s role in improving metabolic health.

### 3.2. Impact of Hemp Seed Cake Protein Concentrate on Éclair Quality

#### 3.2.1. Sensory Evaluation of HPC-Enriched Éclairs

In exploring the impact of hemp seed cake protein concentrate on éclair quality, the appearance and internal structure of samples with varying concentrations of hemp protein concentrate (HPC) were examined: HPC0%, HPC1%, HPC5%, HPC10%, HPC15%, and HPC20%. The top row (Table 5) displays each sample from above, highlighting surface texture and color, while the bottom row reveals the cross-section, illustrating the interior texture and airiness. As HPC concentration increases, variations in crumb structure and crust characteristics become apparent. These visual comparisons provide insight into how HPC levels influence the physical attributes of the éclairs, supporting the sensory evaluation results from the study.

Table 5 visually confirms that higher HPC concentrations lead to noticeable changes, including increased interior volume and enhanced texture. These variations reflect how differing HPC levels influence the éclairs’ physical properties, aligning with the sensory evaluation findings.

A check-all-that-apply (CATA) questionnaire was employed to assess the sensory attributes of six éclair samples differentiated by HPC concentration. Initially, the CATA list included a comprehensive set of 36 sensory terms, but not all terms were relevant for discerning differences among the samples. Following analysis, terms like collapsed, pale, sweet aroma, mild aroma, off-smell, soft exterior, tough exterior, chewy interior, bitter aftertaste, etc., were infrequently selected and removed to refine the sensory evaluation process. This refinement ensured that the remaining 21 significant attributes, determined through Cochran’s Q test (*p* < 0.05), accurately captured the sensory distinctions among the éclair samples. These focused CATA terms facilitated a precise and insightful analysis of the impact of HPC variations on the sensory quality of the éclairs, as illustrated in the plot.

**Figure 1:**
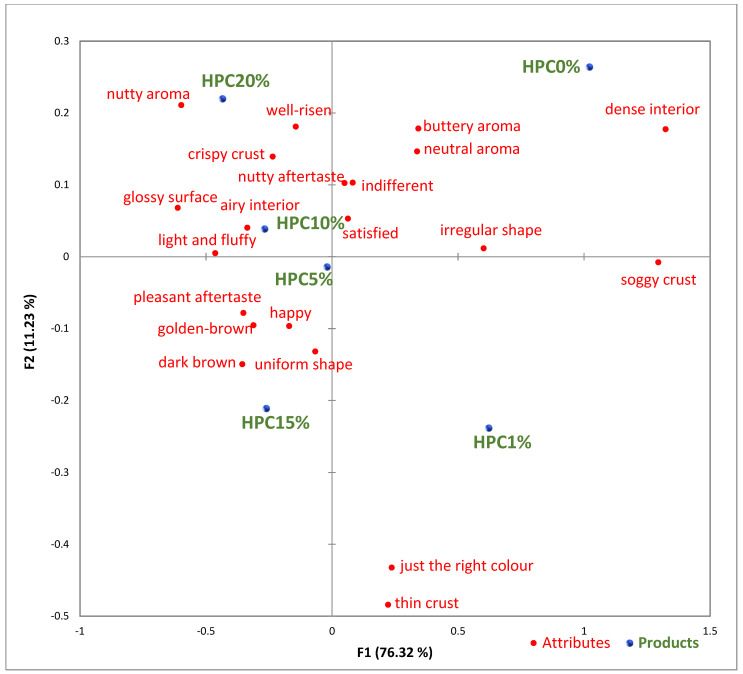
Visualized results of the check-all-that-apply (CATA) analysis of the six éclair samples. HPC0%—control sample; HPC1%—éclair with 1% addition of hempseed cake protein concentrate; HPC5%—éclair with 5% addition of hempseed cake protein concentrate; HPC10%—éclair with 10% addition of hempseed cake protein concentrate; HPC15%—éclair with 15% addition of hempseed cake protein concentrate; HPC20%—éclair with 20% addition of hempseed cake protein concentrate.

Figure 1 highlights sensory attributes associated with varying concentrations of hemp protein concentrate in choux pastry, with distinct profiles for HPC10%, HPC15%, and HPC20%. The HPC10% formulation, associated with attributes like “light and fluffy”, aligns with findings by Xing et al. (2021), who reported that lower levels of protein fortification tend to preserve the delicate structure of baked goods [67]. For HPC15%, linked to “pleasant aftertaste” and “golden-brown” color, the results resonate with Švec and Hrušková (2015), who observed that moderate hemp protein additions enhance bread flavor while maintaining desirable visual characteristics [68]. In contrast, HPC20%, which is characterized by “nutty aroma” and “crispy crust”, reflects findings from Mikulek et al. (2019), where higher protein levels in bread formulations impart strong flavors and improved crust properties, although they might lead to mixed consumer responses due to intensified sensory attributes [69]. Overall, these comparisons with the existing literature reinforce the nuanced impact of hemp protein levels on sensory characteristics, underscoring its potential to enhance the nutritional and sensory qualities of bakery products while highlighting the importance of optimizing concentration levels for consumer acceptance. A positive correlation was established between the visual observations (Table 5) and sensory attributes (Figure 1): The increasing interior volume and improved airiness observed in Table 5 align with the sensory attributes identified through CATA analysis. Specifically, formulations with moderate HPC levels (HPC10%) were associated with descriptors such as “light and fluffy” and “airy interior”, confirming that the structural expansion positively influenced perceived texture and mouthfeel. Conversely, at higher inclusion levels (HPC20%), while the interior volume remained high, sensory responses indicated a shift toward “denser interior” and “chewy texture”, suggesting a limit beyond which increased protein concentration impacts the overall softness and perceived quality of the product. These results reinforce the importance of optimizing HPC levels to balance volume expansion with sensory acceptability.

#### 3.2.2. Proximate Composition, Structural, and Baking Performance of HPC-Enriched Éclairs

The effects of HPC incorporation on the quality attributes of éclairs, indicators such as water activity, acidity, overall volume, and baking loss were analyzed (Table 6).

In assessing the impact of hemp seed cake protein concentrate on éclair quality, specific trends were observed in quality parameters, including a decrease in water activity as the concentration of hemp protein concentrate increased, ranging from 0.618 at HPC0% to 0.565 at HPC20%. This reduction in water activity is consistent with findings from other hemp-fortified products, such as “tsoureki”, a rich-dough baked Greek product [70]. However, statistical analysis did not confirm a significant difference in water activity across formulations at the chosen confidence level (*p* > 0.05), indicating that while a decreasing trend is present, additional validation may be required to establish its statistical relevance. Additionally, baking loss decreased slightly, with values dropping from 26.85% at HPC0% to 24.67% at HPC10%, indicating a reduction in moisture loss. Despite the reduced baking loss and corresponding increase in moisture content (Table 7), the decrease in water activity may be attributed to the ability of the hemp protein concentrate to transform free water into bound water. This binding effect likely enhances the stability and quality of the éclair, underscoring the favorable impact of hemp protein on moisture retention and structural integrity of the final product. Acidity increased progressively from 1.25 in HPC0% to 1.71 degrees in HPC20%, similar to the enhanced acidity reported by Bădărău et al. (2018) in hemp-enriched breads [36].

A notable increase in both the exterior and interior volumes was observed, with exterior volume rising from 42.15 cm^3^ in HPC0% to 51.5 cm^3^ in HPC20%, and interior volume from 18.34 cm^3^ to 38.47 cm^3^. This expansion contrasts with Hrušková et al. (2012), who found decreased loaf volume in hemp breads was attributable to differing effects on structural properties in various baked goods [71]. These outcomes highlight the unique impact of hemp protein concentrate on éclair characteristics, promoting enhanced structural attributes and maintaining moisture retention, providing a stark contrast to its effects in bread formulations, necessitating customized formulations based on product type. The proximate composition of the éclair samples presented in Table 7 further supports these findings by detailing the nutritional and structural parameters mentioned above.

The composition of the éclair samples, as presented in the Table 7, reveals important nutritional parameters that help explain the structural characteristics previously discussed. Notably, the moisture content showed an increasing trend from 31.59% in the HPC0% sample to 34.15% in the HPC20% sample. This rise in moisture content may indicate a greater capacity of the hemp protein to retain moisture, which can contribute to improved texture and structural integrity during baking. Simultaneously, protein content exhibited a significant increase from 8.23% in the HPC0% sample to 11.32% in the HPC20% sample. The increase in protein content correlates with the observed enhancements in the exterior and interior volumes of the éclairs. The higher concentration of protein can lead to better aeration and structural stability, likely due to the protein’s ability to form a cohesive network that supports the overall structure of the product.

The fat content remained consistent across samples, ranging from 5.12% to 5.29%, indicating that the main changes in the quality of the éclairs are attributed to the variations in protein and moisture content rather than fat. On the other hand, the carbohydrate content showed a gradual decline, starting at 54.89% in the HPC0% sample and decreasing to 49.39% in the HPC20% sample.

The Pearson correlation analysis was conducted to investigate the relationships between the addition of hemp seed protein concentrate and key physico-chemical indicators of the product. This approach allows for identifying significant dependencies and interactions among variables, providing insights into how protein concentration impacts product structure, stability, and quality.

**Figure 2:**
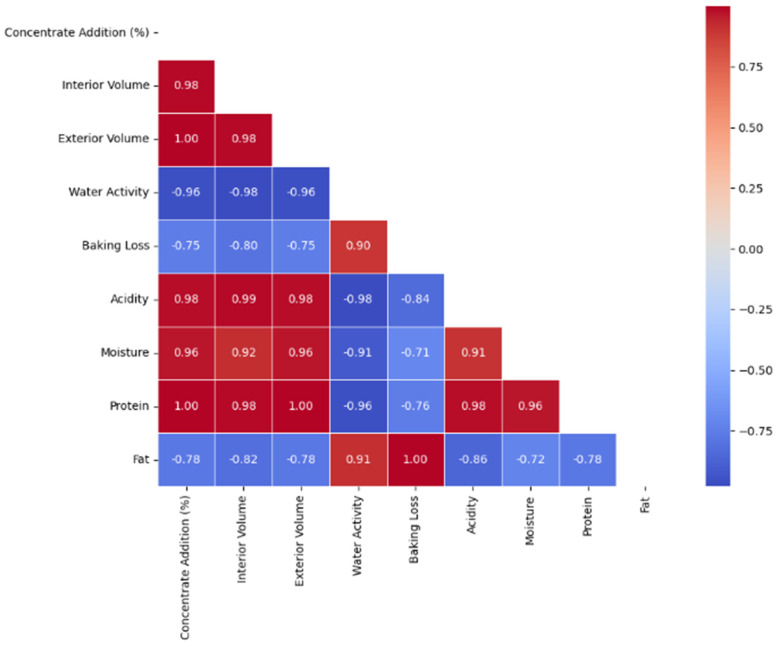
Pearson correlation coefficient between different measures and indicators.

As shown in Figure 2, the addition of hemp seed protein concentrate (% concentrate addition) significantly influences key physico-chemical indicators. A strong positive correlation with interior volume (r = 0.98) and exterior volume (r = 1.00) highlights its role in enhancing product structure, likely by reinforcing the protein network. Similarly, protein content (r = 1.00) and moisture (r = 0.96) show strong relationships with concentrate addition, indicating improved water retention and texture. Conversely, water activity decreases sharply with increasing protein concentrations (r = −0.96), reflecting reduced free water due to a denser structure. This reduction also correlates with lower baking loss (r = −0.75), suggesting better stability during processing. The positive correlation of acidity with moisture (r = 0.91) and protein content (r = 0.98) points to potential benefits in terms of preservation and sensory properties. While fat content shows moderate negative correlations with most variables (e.g., r = −0.78 with interior volume), its impact appears secondary to the protein’s dominant influence.

To assess the broader nutritional implications of incorporating hemp seed cake protein concentrate, the contribution of this ingredient was determined analytically, considering the literature sources that describe the amino acid profiles of other raw materials such as wheat flour, eggs, milk, and butter. For the hemp concentrate, data obtained from research and presented in Table 4 were used. These findings highlight the increase in essential and non-essential amino acids in the final products (Table 8), underscoring the significant nutritional benefits of HPC enrichment.

The amino acid content in baked choux pastry with increasing hemp protein concentrate (HPC) levels shows a clear nutritional enhancement. Essential amino acids like threonine and isoleucine increase substantially—from 0.30 g and 0.32 g in HPC0% to 0.48 g and 0.51 g in HPC20%, respectively. These amino acids are essential for muscle protein synthesis and immune system support, emphasizing the dietary benefits of HPC-enriched products [76,77,78].

Similarly, non-essential amino acids such as aspartic and glutamic acids rise notably (aspartic acid from 0.62 g to 0.81 g and glutamic acid from 0.79 g to 1.50 g), which are vital for cellular metabolism and neurotransmission. Methionine, increasing from 0.18 g to 0.23 g, plays an essential role in detoxification and metabolic processes. The presence of gamma-aminobutyric acid (GABA) with higher HPC levels adds potential benefits for reducing stress and promoting neural health. Research by House et al. (2010) and Rusu et al. (2021) supports these findings, highlighting similar amino acid profile improvements in hemp-fortified foods [79,80]. These enhancements contribute to better overall health, reflecting the potential of HPC to provide a balanced and nutritious dietary option in bakery products. Other researchers have explored the fortification of bakery products with various plant-based protein sources, yielding both similar and divergent findings. For example, studies on soy and pea protein concentrates have shown comparable enhancements in essential amino acid profiles, particularly in leucine and lysine content, which align with our findings for hemp cake protein concentrate [81]. However, some studies have reported lower digestibility or less favorable amino acid balances when using certain plant proteins, which contrasts with the balanced amino acid profile observed in hemp seed protein [82].

Conversely, antagonistic results have been noted in research using wheat gluten or rice protein, where deficiencies in essential amino acids like lysine and threonine were more pronounced [83,84]. These discrepancies highlight the importance of selecting appropriate protein sources depending on the nutritional goals of the fortified food product.

#### 3.2.3. Color Parameters of HPC-Enriched Éclairs

To evaluate the impact of hemp seed cake protein concentrate on the éclairs’ color, parameters such as crust and crumb color were measured (Table 9).

The results indicate a noticeable decrease in lightness (L*) for both crust and crumb as HPC concentration increased, with crust L* values dropping from 89.62 in HPC0% to 82.38 in HPC20% and crumb L* values from 93.78 to 88.41. This trend aligns with findings by Lazou et al. (2023), who observed similar darkening effects in hemp-enriched baked goods, likely due to Maillard reactions intensified by hemp compounds [85].

The a* values, indicating redness, declined in the crust, suggesting a reduction in red hues with higher HPC concentrations. This decrease is consistent with studies on plant-based protein supplements altering typical browning patterns [86]. Meanwhile, b* values, representing yellowness, also decreased, particularly in the crumb, from 5.63 in HPC0% to 4.34 in HPC20%. This reduction in yellowness echoes reports by Lučan Čolić et al. (2024) on the influence of hemp ingredients on color dynamics [87].

The total color difference (Δ*E*) increased with more HPC, showing significant visual divergence from the control, notably in crust color with Δ*E* reaching 9.17 at HPC20%. These findings suggest that incorporating hemp protein concentrate significantly influences the color, reflecting protein and fiber interactions, and aligns with broader research on natural ingredient impacts on baking outcomes.

## 4. Conclusions

The incorporation of hemp seed cake protein concentrate (HPC) into choux pastry formulations, specifically éclairs, provides significant benefits in terms of nutritional enhancement and sensory characteristics. Initially, the protein concentrate was obtained with a total protein content of 74.15%, featuring a well-balanced amino acid profile that included essential amino acids such as leucine at 8.17 g/100 g protein and isoleucine at 5.56 g/100 g protein, highlighting its potential as a superior protein source.

In the formulation of éclairs, the results demonstrated a notable increase in overall protein content from 8.23% in the control sample (HPC0%) to 11.32% in the HPC20% formulation, accompanied by a significant rise in moisture content from 31.59% to 34.15%. These changes contributed to enhanced structural attributes, with exterior and interior volumes increasing from 42.15 cm^3^ to 51.5 cm^3^ and from 18.34 cm^3^ to 38.47 cm^3^, respectively. The Pearson correlation analysis further confirmed strong positive relationships between protein addition, volume, and moisture, while also revealing inverse correlations with water activity and baking loss, underscoring the role of hemp protein concentrate in optimizing the structural and physico-chemical properties of the éclairs.

Amino acid composition analysis further revealed substantial increases in key amino acids in the éclairs: the content of threonine increased from 0.30 g in the HPC0% sample to 0.48 g in the HPC20% sample, and isoleucine rose from 0.32 g to 0.51 g, emphasizing the nutritional benefits of HPC enrichment.

Sensory evaluations indicated that formulations with up to 10% HPC achieved optimal profiles, marked by desirable features such as “light and fluffy” texture and “pleasant aftertaste”.

In terms of color, increasing HPC levels led to a progressive darkening of both crust and crumb, with lightness (*L* value) decreasing from 89.62 in HPC0% to 82.38 in HPC20%. Although moderate HPC additions contributed to an appealing golden-brown crust, the excessive color change at 20% HPC (Δ*E* = 9.17) could influence consumer acceptance.

Considering all analyzed parameters—including protein enrichment, amino acid composition, structural properties, moisture retention, color stability, and sensory acceptability—the optimal HPC inclusion level for enhancing both the nutritional and qualitative properties of éclairs was determined to be 10%.

Beyond its application in pastry products, our findings have broader implications for the food industry, particularly in the development of functional, high-protein bakery and plant-based food formulations. The scalability of HPC as an ingredient is supported by its natural abundance as a byproduct of hemp oil extraction, aligning with sustainable food production practices and circular economy principles. Given the increasing consumer demand for clean-label and plant-derived proteins, HPC has the potential for wider adoption in various food categories, including protein-enriched bread, snack formulations, or even meat analogues, where it could improve the protein quality while maintaining desirable textural properties.

## Figures and Tables

**Table 1:** Choux pastry formulations.

Raw Materials	Hemp Seed Cake Protein Concentrate, % (% Replacement of Wheat Flour)					
	0%	1%	5%	10%	15%	20%
Wheat flour, g	200	198	190	180	170	160
Hemp seed cake protein concentrate, g	0	2	10	20	30	40
Eggs, g	330	330	330	330	330	330
Milk, mL	200	200	200	200	200	200
Water, mL	200	200	200	200	200	200
Butter, g	188	188	188	188	188	188
Sugar, g	10	10	10	10	10	10
Salt, g	3	3	3	3	3	3
Total, g	1131	1131	1131	1131	1131	1131

**Table 2:** CATA attributes for éclair sensory analysis.

Attribute Category	CATA Attributes
Appearance	Uniform shape, cracked surface, glossy surface, well-risen, irregular shape, collapsed
Color	Golden-brown, pale, dark brown, just the right color
Aroma	Buttery aroma, baked aroma, neutral aroma, nutty aroma, sweet aroma, mild aroma, off-smell
Texture (Exterior)	Crispy crust, thin crust, soft exterior, tough exterior, soggy crust
Texture (Interior)	Airy interior, light and fluffy, dense interior, moist interior, dry interior, sticky, chewy interior, smooth texture, coarse texture
Aftertaste	Pleasant aftertaste, nutty aftertaste, bitter aftertaste, no aftertaste, lingering sweetness
Emotions	Happy, satisfied, excited, curious, indifferent, disappointed, frustrated, surprised

**Table 3:** Physicochemical indicators of the wheat flour and hemp seed cake protein concentrate.

Indicator	Wheat Flour	Hemp (*Cannabis sativa* L.) Seed Cake Protein Concentrate
Total protein content, % d.m	10.3 *	74.15 ± 0.14
Carbohydrates, % d.m	71.9 *	4.11 ± 0.03
Total dietary fiber content, % d.m.	2.15 ± 0.04 ^a^	18.47 ± 0.07 ^b^
Fat content, % d.m.	1.08 *	0.45 ± 0.02
Moisture content, %	13.35 ± 0.07 ^b^	3.12 ± 0.06 ^a^
Ash content, % d.m.	0.84 ± 0.01 ^a^	2.14 ± 0.04 ^b^
Acidity, degrees	2.83 ± 0.06 ^b^	1.16 ± 0.02 ^a^

Results indicate the mean value of three independent assays and are expressed as mean ± standard deviation (SD), in each raw different letters ^a,b^ mean significant differences (*p* ˂ 0.05). * The values (%) were taken from the product label and converted into % d.m.

**Table 4:** Comparison of amino acids content of the hemp seed cake protein concentrate with protein pattern of FAO.

Amino Acid	Hemp Seed Cake Protein Concentrate			FAO/WHO Standard, mg/g [42]
	Amino Acid Content, g/kg	g Amino Acid/100 g Protein	*AAS*, %	
Aspartic acid	44.51 ± 0.16	5.94 ± 0.04		
Threonine	40.33 ± 0.12	5.38 ± 0.03	234.04	2.3
Serine	51.11 ± 0.09	6.82 ± 0.02		
Glutamic acid	151.07 ± 0.24	20.16 ± 0.04		
Proline	32.14 ± 0.07	4.29 ± 0.03		
Glycine	36.07 ± 0.05	4.81 ± 0.07		
Alanine	44.81 ± 0.18	5.98 ± 0.06		
Valine	20.62 ± 0.17	2.75 ± 0.02	70.57	3.9
Cysteine	3.27 ± 0.05	0.44 ± 0.01	19.84	2.2
Methionine	11.79 ± 0.04	1.57 ± 0.02		
Isoleucine	41.64 ± 0.13	5.56 ± 0.05	185.26	3.0
Leucine	61.19 ± 0.22	8.17 ± 0.06	138.42	5.9
Tyrosine	20.05 ± 0.16	2.68 ± 0.02	70.42	3.8
Phenylalanine	47.25 ± 0.18	6.31 ± 0.06		
Gamma-aminobutyric acid (GABA)	2.72 ± 0.03	0.36 ± 0.01		
Lysine	29.52 ± 0.12	3.94 ± 0.03	87.56	4.5
Histidine	22.17 ± 0.14	2.96 ± 0.02	197.27	1.5
Arginine	81.84 ± 0.27	10.92 ± 0.07		
Ammonia	7.13 ± 0.13	0.95 ± 0.01		
Total free amino acids	742.1 ± 0.46	99.05 ± 0.11		
Total nitrogen metabolism indices	749.23 ± 0.54	100.00 ± 0.14		
Total essential amino acids	356.35 ± 0.35	47.56 ± 0.12		
Total non-essential amino acids	383.03 ± 0.29	51.12 ± 0.22		
Total immunoactive amino acids	272.25 ± 0.24	36.34 ± 0.16		
Total glycogenic amino acids	539.73 ± 0.47	72.04 ± 0.18		
Total ketogenic amino acids	199.65 ± 0.31	26.65 ± 0.04		
Total proteinogenic amino acids	739.38 ± 0.22	98.69 ± 0.13		
Total sulfur-containing amino acids	15.06 ± 0.07	2.01 ± 0.02		

Results indicate the mean value of three independent assays and are expressed as mean ± standard deviation (SD).

**Table 5:** Éclair samples view from above and in section.

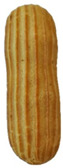	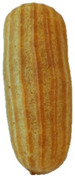	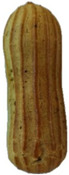
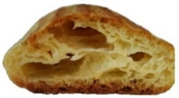	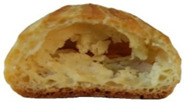	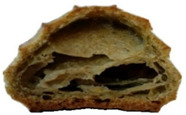
**HPC0%**	**HCP1%**	**HCP5%**
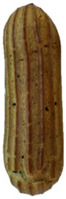	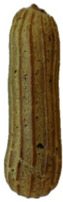	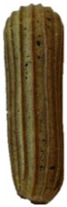
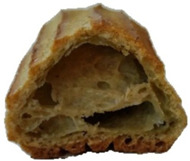	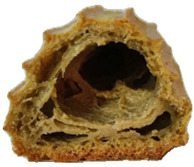	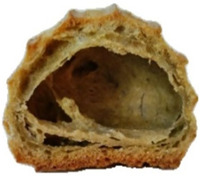
**HCP10%**	**HCP15%**	**HCP20%**

HPC0%—control sample; HPC1%—éclair with 1% addition of hempseed cake protein concentrate; HPC5%—éclair with 5% addition of hempseed cake protein concentrate; HPC10%—éclair with 10% addition of hempseed cake protein concentrate; HPC15%—éclair with 15% addition of hempseed cake protein concentrate; HPC20%—éclair with 20% addition of hempseed cake protein concentrate.

**Table 6:** Quality parameters of éclair samples.

Sample	Water Activity (aw)	Acidity, Degrees	Exterior Volume, (cm^3^)	Interior Volume, (cm^3^)	Baking Loss, (%)
HPC0%	0.618 ± 0.005 ^a^	1.25 ± 0.05 ^a^	42.15 ± 0.24 ^a^	18.34 ± 0.15 ^a^	26.85 ± 0.20 ^c^
HPC1%	0.604 ± 0.003 ^a^	1.31 ± 0.04 ^ab^	42.62 ± 0.19 ^ab^	19.63 ± 0.11 ^ab^	25.50 ± 0.19 ^b^
HPC5%	0.602 ± 0.002 ^a^	1.42 ± 0.05 ^b^	44.49 ± 0.21 ^b^	24.79 ± 0.09 ^b^	25.95 ± 0.22 ^b^
HPC10%	0.578 ± 0.004 ^a^	1.58 ± 0.06 ^c^	46.83 ± 0.17 ^c^	31.24 ± 0.12 ^c^	24.67 ± 0.17 ^a^
HPC15%	0.567 ± 0.005 ^a^	1.62 ± 0.05 ^cd^	49.16 ± 0.14 ^d^	37.28 ± 0.11 ^d^	24.85 ± 0.19 ^a^
HPC20%	0.565 ± 0.002 ^a^	1.71 ± 0.06 ^d^	51.5 ± 0.16 ^e^	38.47 ± 0.08 ^e^	24.95 ± 0.18 ^a^

Results indicate the mean value of three independent assays and are expressed as mean ± standard deviation (SD), in each column different letters ^a–e^ mean significant differences (*p* ˂ 0.05).

**Table 7:** Proximate composition of éclair samples, %.

Sample	Moisture	Proteins	Fat	Carbohydrate *
HPC0%	31.59 ± 0.18 ^ab^	8.23 ± 0.08 ^a^	5.29 ± 0.05 ^a^	54.89 ± 0.15 ^c^
HPC1%	31.94 ± 0.14 ^ab^	8.39 ± 0.13 ^a^	5.19 ± 0.04 ^a^	54.48 ± 0.14 ^c^
HPC5%	31.75 ± 0.31 ^ab^	9.01 ± 0.11 ^ab^	5.22 ± 0.07 ^a^	54.02 ± 0.16 ^c^
HPC10%	32.46 ± 0.12 ^b^	9.79 ± 0.08 ^b^	5.12 ± 0.03 ^a^	52.63 ± 0.13 ^b^
HPC15%	33.24 ± 0.17 ^a^	10.55 ± 0.06 ^c^	5.13 ± 0.04 ^a^	51.08 ± 0.15 ^a^
HPC20%	34.15 ± 0.24 ^c^	11.32 ± 0.11 ^d^	5.14 ± 0.06 ^a^	49.39 ± 0.12 ^a^

Results indicate the mean value of three independent assays and are expressed as mean ± standard deviation (SD), in each raw different letters ^a–d^ mean significant differences (*p* ˂ 0.05). * calculated by difference.

**Table 8:** Contribution of hemp seed cake protein concentrate to the amino acid content in éclairs (mg/100 g product).

Amino Acid	HPC0%	HPC1%	HPC5%	HPC10%	HPC15%	HPC20%
Aspartic acid	0.62	0.63	0.67	0.71	0.76	0.81
Threonine	0.30	0.31	0.35	0.39	0.44	0.48
Serine	0.40	0.42	0.46	0.52	0.58	0.64
Glutamic acid	0.79	0.82	0.96	1.14	1.32	1.50
Proline	0.33	0.33	0.36	0.40	0.43	0.47
Glycine	0.25	0.26	0.29	0.33	0.37	0.41
Alanine	0.30	0.31	0.35	0.41	0.46	0.51
Valine	0.33	0.34	0.36	0.38	0.41	0.43
Cysteine	0.08	0.08	0.08	0.09	0.09	0.09
Methionine	0.18	0.18	0.19	0.21	0.22	0.23
Isoleucine	0.32	0.33	0.36	0.41	0.46	0.51
Leucine	0.56	0.57	0.63	0.70	0.77	0.83
Tyrosine	0.26	0.27	0.28	0.31	0.33	0.35
Phenylalanine	0.37	0.38	0.42	0.48	0.53	0.58
Gamma-amino butyric acid (GABA)	0.00	0.00	0.00	0.01	0.01	0.01
Lysine	0.45	0.46	0.49	0.52	0.55	0.58
Histidine	0.18	0.19	0.21	0.23	0.25	0.28
Arginine	0.41	0.43	0.50	0.59	0.69	0.78

Note: Data for other ingredients (i.e., wheat flour, eggs, milk, butter) than hemp seed cake concentrate were taken from the literature and calculated for éclair formulations [72,73,74,75].

**Table 9:** Color parameters of éclair samples with hemp seed cake protein concentrate.

	HPC0%	HCP1%	HCP5%	HCP10%	HCP15%	HCP20%
Crust color						
L*	89.62 ± 0.24 ^e^	88.47 ± 0.45 ^d^	87.64 ± 0.13 ^cd^	87.25 ± 0.24 ^c^	86.14 ± 0.16 ^b^	82.38 ± 0.21 ^a^
a*	3.65 ± 0.23 ^cd^	3.24 ± 0.18 ^c^	2.76 ± 0.14 ^bc^	2.36 ± 0.17 ^b^	1.48 ± 0.13 ^ab^	1.27 ± 0.19 ^a^
b*	11.26 ± 0.24 ^d^	10.42 ± 0.17 ^c^	7.25 ± 0.14 ^b^	6.86 ± 0.19 ^ab^	6.43 ± 0.21 ^a^	6.15 ± 0.14 ^a^
Δ*E*		1.48 ± 0.13 ^a^	4.56 ± 0.14 ^bc^	5.16 ± 0.17 ^c^	6.34 ± 0.11 ^d^	9.17 ± 0.12 ^e^
Crumb color						
L*	93.78 ± 0.15 ^d^	93.42 ± 0.12 ^c^	90.59 ± 0.09 ^bc^	89.76 ± 0.17 ^b^	89.22 ± 0.08 ^ab^	88.41 ± 0.16 ^a^
a*	1.56 ± 0.17 ^a^	1.47 ± 0.14 ^a^	1.38 ± 0.16 ^a^	1.32 ± 0.15 ^a^	1.26 ± 0.12 ^a^	1.21 ± 0.11 ^a^
b*	5.63 ± 0.14 ^c^	5.52 ± 0.16 ^bc^	5.41 ± 0.07 ^b^	5.14 ± 0.09 ^b^	4.74 ± 0.16 ^ab^	4.34 ± 0.18 ^a^
Δ*E*		0.39 ± 0.08 ^a^	3.20 ± 0.14 ^b^	4.06 ± 0.18 ^bc^	4.66 ± 0.13 ^c^	5.54 ± 0.15 ^d^

Results indicate the mean value of three independent assays and are expressed as mean ± standard deviation (SD), in each raw different letters ^a–e^ mean significant differences (*p* ˂ 0.05). HPC0%—control sample; HPC1%—éclair with 1% addition of hempseed cake protein concentrate; HPC5%—éclair with 5% addition of hempseed cake protein concentrate; HPC10%—éclair with 10% addition of hempseed cake protein concentrate; HPC15%—éclair with 15% addition of hempseed cake protein concentrate; HPC20%—éclair with 20% addition of hempseed cake protein concentrate.

## Data Availability

The original contributions presented in the study are included in the article, further inquiries can be directed to the corresponding author.
